# Redetermination of *cis*-bis­(ethyl­ene­diamine-κ^2^ *N*,*N*′)bis­(nitrito-κ*N*)cobalt(III) (ethyl­enediamine-κ^2^ *N*,*N*′)tetra­kis­(nitrito-κ*N*)cobaltate(III) monohydrate

**DOI:** 10.1107/S1600536812050325

**Published:** 2012-12-15

**Authors:** Robert A. Burrow, Juliano R. de Menezes Vicenti

**Affiliations:** a Laboratório de Materiais Inorgânicos, Universidade Federal de Santa Maria, 97105–900 Santa Maria, RS, Brazil

## Abstract

The structure of the title compound, [Co(NO_2_)_2_(NH_2_CH_2_CH_2_NH_2_)_2_][Co(NO_2_)_4_(NH_2_CH_2_CH_2_NH_2_)]·H_2_O, was redetermined with a modern CCD-equipped diffractometer. In comparison with the original determination based on photographic data [Kushi *et al.* (1976 ▶). *Inorg. Nucl. Chem. Lett*. **12**, 629–633], the current study allows the location of reliable postions for the H atoms and thus leads to better understanding of the inter­ionic and inter­molecular inter­actions. The crystal structure consists of an octa­hedrally coordinated cationic Co^III^ complex ion, an octa­hedrally coordinated anionic Co^III^ complex ion and a lattice water mol­ecule. The complex cation, complex anion and lattice water mol­ecule are connected by an intricate network of O—H⋯O and N—H⋯O hydrogen bonds, forming a three-dimensional structure.

## Related literature

For background to Co^III^ complexes, see: Angelici (1969 ▶); Bernal (1985 ▶); Bernal & Kauffman (1987 ▶); Murmann (1955 ▶). For a previous report of the crystal structure of the title compound, see: Kushi *et al.* (1976 ▶). For synthetic details, see: Bailor & Rollinson (1946 ▶); Sharrock (1980 ▶). For a description of the Cambridge Structural Database, see: Allen (2002 ▶).
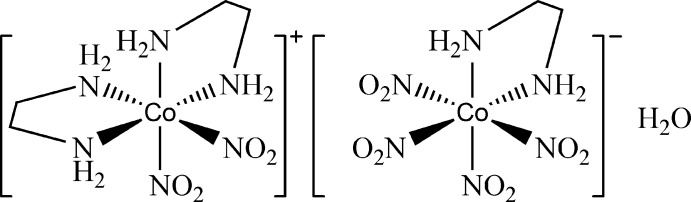


## Experimental

### 

#### Crystal data

[Co(NO_2_)_2_(C_2_H_8_N_2_)_2_][Co(NO_2_)_4_(C_2_H_8_N_2_)]·H_2_O
*M* *_r_* = 592.25
Monoclinic, 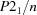

*a* = 14.7580 (5) Å
*b* = 6.7060 (2) Å
*c* = 20.6845 (7) Å
β = 96.969 (2)°
*V* = 2031.96 (11) Å^3^
*Z* = 4
Mo *K*α radiation
μ = 1.73 mm^−1^
*T* = 100 K
0.40 × 0.15 × 0.05 mm

#### Data collection

Bruker X8 Kappa APEXII diffractometer
Absorption correction: numerical (*SADABS*; Bruker, 2012 ▶) *T*_min_ = 0.692, *T*_max_ = 0.925
63576 measured reflections
6280 independent reflections
4801 reflections with *I* > 2σ(*I*)
*R*_int_ = 0.073

#### Refinement

*R*[*F* ^2^ > 2σ(*F* ^2^)] = 0.035
*wR*(*F* ^2^) = 0.087
*S* = 1.07
6280 reflections
340 parameters
H atoms treated by a mixture of independent and constrained refinement
Δρ_max_ = 0.72 e Å^−3^
Δρ_min_ = −0.84 e Å^−3^

### 

Data collection: *APEX2* (Bruker, 2012 ▶); cell refinement: *SAINT* (Bruker, 2012 ▶); data reduction: *SAINT*; program(s) used to solve structure: *SHELXS97* (Sheldrick, 2008 ▶); program(s) used to refine structure: *SHELXL97* (Sheldrick, 2008 ▶); molecular graphics: *DIAMOND* (Brandenburg, 2009 ▶); software used to prepare material for publication: *publCIF* (Westrip, 2010 ▶).

## Supplementary Material

Crystal structure: contains datablock(s) global, I. DOI: 10.1107/S1600536812050325/wm2706sup1.cif

Structure factors: contains datablock(s) I. DOI: 10.1107/S1600536812050325/wm2706Isup2.hkl

Additional supplementary materials:  crystallographic information; 3D view; checkCIF report

## Figures and Tables

**Table 1:** Hydrogen-bond geometry (Å, °)

*D*—H⋯*A*	*D*—H	H⋯*A*	*D*⋯*A*	*D*—H⋯*A*
N11—H11*A*⋯O11^i^	0.83 (3)	2.26 (3)	3.048 (2)	158 (2)
N11—H11*B*⋯O23^ii^	0.79 (3)	2.23 (3)	2.991 (2)	163 (2)
N11—H11*B*⋯O13^iii^	0.79 (3)	2.57 (2)	2.966 (2)	113 (2)
N12—H12*A*⋯O24^iv^	0.86 (3)	2.22 (3)	3.060 (2)	164 (2)
N12—H12*B*⋯O12^v^	0.80 (3)	2.38 (3)	3.030 (2)	139 (2)
N13—H13*A*⋯O22^vi^	0.84 (3)	2.42 (3)	3.186 (2)	152 (2)
N13—H13*B*⋯O13^iii^	0.87 (3)	2.47 (3)	3.206 (2)	143 (2)
N14—H14*A*⋯O27^ii^	0.94 (3)	2.12 (3)	3.038 (2)	164 (2)
N14—H14*B*⋯O24^iv^	0.85 (2)	2.42 (2)	3.060 (2)	132 (2)
N21—H21*A*⋯O28^vii^	0.82 (3)	2.56 (3)	3.185 (2)	134 (2)
N22—H22*A*⋯O27^viii^	0.83 (2)	2.18 (3)	2.981 (2)	164 (2)
N22—H22*B*⋯O1	0.89 (2)	2.16 (3)	2.967 (2)	151 (2)
O1—H1*A*⋯O26^iv^	0.84 (3)	2.19 (3)	2.953 (2)	152 (3)
O1—H1*A*⋯O24^iv^	0.84 (3)	2.53 (3)	3.081 (2)	124 (2)
O1—H1*B*⋯O25^viii^	0.82 (3)	2.07 (3)	2.866 (2)	162 (3)

Symmetry codes: (i) 
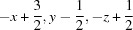
; (ii) 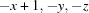; (iii) 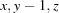; (iv) 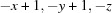; (v) 
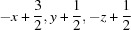
; (vi) 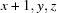; (vii) 
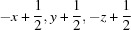
; (viii) 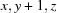.

**Fig. 1.**
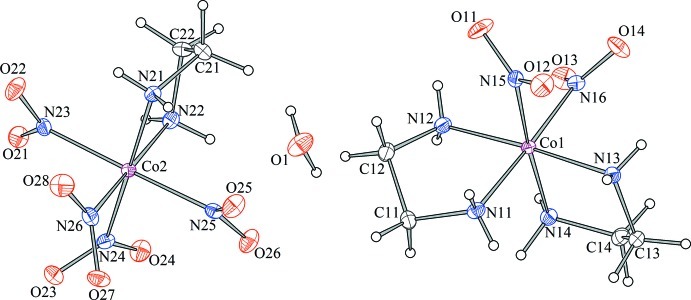
The molecular structure of (I), with 50% probability ellipsoids, showing the atomic numbering scheme.

**Fig. 2.**
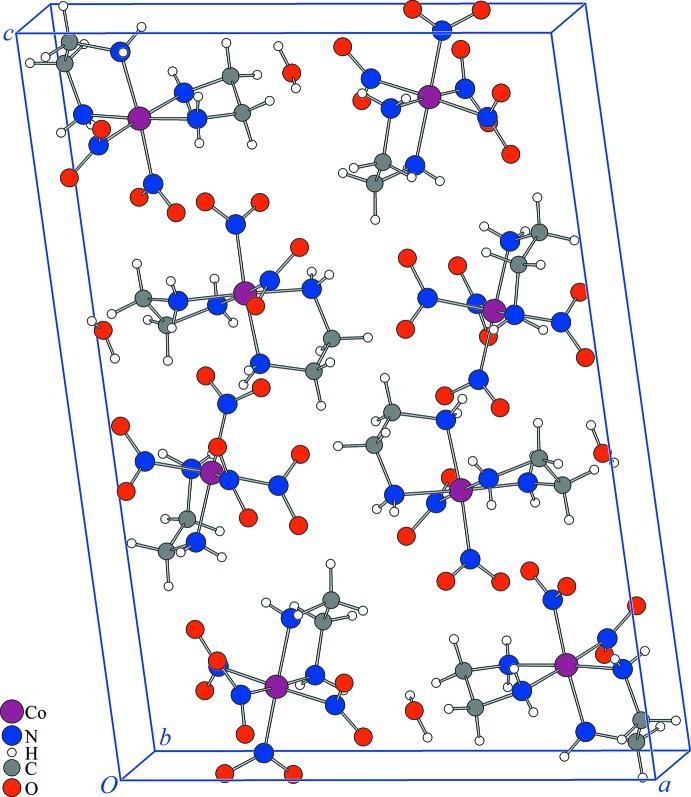
The packing diagram of (I) projected along the b axis.

**Fig. 3.**
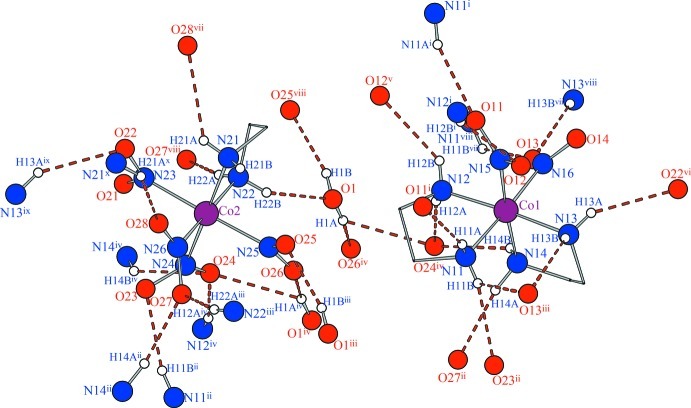
The hydrogen bonding interactions in the crystal structure of (I) shown as dashed red lines. [Symmetry codes: (i) -x + 3/2, y - 1/2, -z + 1/2; (ii) -x + 1, -y, -z; (iii) x, y - 1, z; (iv) -x + 1, -y + 1, -z; (v) -x + 3/2, y + 1/2, -z + 1/2; (vi) x + 1, y, z; (vii) -x + 1/2, 320 y + 1/2, -z + 1/2; (viii) x, y + 1, z; (ix) x - 1, y, z; (x) -x + 1/2, y - 1/2, -z + 1/2.]

## supplementary crystallographic information

### Comment

Cobalt(III) complexes are classical examples in undergraduate inorganic experimental laboratories due to their ease of preparation and great stability (Angelici, 1969). The ethylenediamine complex *cis*-[bis(ethylenediamine-kN,*N*')dinitrito-κ-N-cobalt(III)] chloride is of particular interest due to its spontaneous resolution upon crystallization (Murmann, 1955; Bernal, 1985; Bernal & Kauffman, 1987). In an attempt to synthesize this compound, crystals of the title compound *cis*-[Co(NH_2_CH_2_CH_2_NH_2_)_2_(NO_2_)_2_] [Co(NH_2_CH_2_CH_2_NH_2_)(NO_2_)_4_].H_2_O, (I), were obtained instead.

Although the crystal structure of the compound (I) has been determined previously from visually estimated photographic data (Kushi *et al.*, 1976), it is of rather low quality (*R* = 0.13) compared to today's standard, and more importantly the extensive hydrogen bonding was not noted, in part due to the inability to locate the H atoms of the water molecule. In addition, the atomic coordinates have not been deposited with the Cambridge Structure Database (CSD; Allen, 2002) and hence are not available in the public domain. We report here the redetermination of the crystal structure at 100 K with data measured up to 30 ° in θ.

The crystal structure of (I) is centrossymmetric with a racemic mixture of the Δ and Λ isomers of the complex cation, the complex anion and a lattice water molecule. In the complex cation, the two ethylenediamine ligands chelate to the Co^III^ ion, and the nitrito ligands bond *via* their N atoms to form an approximate octahedral coordination geometry. The complex anion is similar, with one ethylenediamine ligand and four nitrito ligands bonded to the central metal cation. The Co—N distances to the ethylenediamine ligands are similar in the two ion complexes, varying between 1.9141 (17) Å and 1.9811 (17) Å. This range is within the distribution for similar complexes with octahedrally coordinated Co(III) found in the CSD (Allen, 2002; version 5.33 as of November, 2011 with Feb., 2011, Mar., 2012 & May, 2012 updates], *viz* 1.97 (2) Å for 756 distances. There is a slight *trans* influence in the the cation complex where the Co—N distance is marginally longer (by *ca* 0.02 Å) for the N atoms *trans* to the nitrito ligands. The Co—N distances for the nitrito ligands show a larger variation with shorter distances in the complex cation, 1.9141 (17) & 1.9177 (16) Å, than those in the complex anion. The latter shows a stronger *trans* influence with the Co—N distances *trans* to the N atoms of the nitrito ligands longer (1.9413 (17) & 1.9502 (16) Å) than the Co—N distance *trans* to the ethylenediamine ligand (1.9215 (17) & 1.9240 (17) Å).

The packing diagram (Fig. 2), shows alternating columns of complex cations and anions in the crystallographic *a* direction. The lattice water molecule is located between the complex cation and anion. There is an intricate three-dimensional network of hydrogen bonding interactions between the NH_2_ groups and O atoms of nitrito ligands of neighboring ions and also of the lattice water molecule, which forms hydrogen bonds to four complex anions (Fig. 3; Table 1).

### Experimental

The title compound was synthesized *via* the chloride, prepared following the procedure of Bailor & Rollinson (1946) with the hydrogen peroxide oxidation modification of Sharrock (1980), followed by subsitution of of the chloride ligand by nitrito ligand (Bernal, 1985). Yellow crystals suitable for single-crystal *X* ray diffraction were formed by slow evaporation of the reaction mixture as room temperature.

### Refinement

The H atoms on N atoms and in the lattice water molecule were found in a difference Fourier map and their positions allowed to refine freely while isotropic displacement factors were set to 1.2 times those of the N atoms or to 1.5 of that the O atom. The H atoms on the ethylene C atoms were positioned geometrically and allowed to ride on their parent atoms, with C—H bond lengths of 0.99 Å and isotropic displacement parameters equal to 1.2 times *U*_eq_ of the parent atom.

### Crystal data

**Table:**

[Co(NO_2_)_2_(C_2_H_8_N_2_)_2_][Co(NO_2_)_4_(C_2_H_8_N_2_)]·H_2_O	*F*(000) = 1216
*M**_r_* = 592.25	*D*_x_ = 1.936 Mg m^−^^3^
Monoclinic, *P*2_1_/*n*	Mo *K*α radiation, λ = 0.71073 Å
*a* = 14.7580 (5) Å	Cell parameters from 7429 reflections
*b* = 6.7060 (2) Å	θ = 2.8–30.0°
*c* = 20.6845 (7) Å	µ = 1.73 mm^−^^1^
β = 96.969 (2)°	*T* = 100 K
*V* = 2031.96 (11) Å^3^	Plate, light yellow
*Z* = 4	0.40 × 0.15 × 0.05 mm

### Data collection

**Table:**

Bruker X8 Kappa APEXII diffractometer	6280 independent reflections
Radiation source: sealed ceramic X ray tube, Siemens KFF	4801 reflections with *I* > 2σ(*I*)
Graphite crystal monochromator	*R*_int_ = 0.073
Detector resolution: 8.3333 pixels mm^-1^	θ_max_ = 30.7°, θ_min_ = 2.8°
0.5 ° ω & φ scans	*h* = −21→21
Absorption correction: numerical (*SADABS*; Bruker, 2012)	*k* = −9→9
*T*_min_ = 0.692, *T*_max_ = 0.925	*l* = −29→29
63576 measured reflections	

### Refinement

**Table:**

Refinement on *F*^2^	Primary atom site location: structure-invariant direct methods
Least-squares matrix: full	Secondary atom site location: difference Fourier map
*R*[*F*^2^ > 2σ(*F*^2^)] = 0.035	Hydrogen site location: inferred from neighbouring sites
*wR*(*F*^2^) = 0.087	H atoms treated by a mixture of independent and constrained refinement
*S* = 1.07	*w* = 1/[σ^2^(*F*_o_^2^) + (0.0384*P*)^2^ + 0.7699*P*] where *P* = (*F*_o_^2^ + 2*F*_c_^2^)/3
6280 reflections	(Δ/σ)_max_ < 0.001
340 parameters	Δρ_max_ = 0.72 e Å^−^^3^
0 restraints	Δρ_min_ = −0.84 e Å^−^^3^

### Special details

**Table:**

Experimental. The data collection was performed under a cold nitritogen flow at 100 K.
Geometry. All e.s.d.'s (except the e.s.d. in the dihedral angle between two l.s. planes) are estimated using the full covariance matrix. The cell e.s.d.'s are taken into account individually in the estimation of e.s.d.'s in distances, angles and torsion angles; correlations between e.s.d.'s in cell parameters are only used when they are defined by crystal symmetry. An approximate (isotropic) treatment of cell e.s.d.'s is used for estimating e.s.d.'s involving l.s. planes.
Refinement. Refinement of *F*^2^ against ALL reflections. The weighted *R*-factor *wR* and goodness of fit *S* are based on *F*^2^, conventional *R*-factors *R* are based on *F*, with *F* set to zero for negative *F*^2^. The threshold expression of *F*^2^ > σ(*F*^2^) is used only for calculating *R*-factors(gt) *etc*. and is not relevant to the choice of reflections for refinement. *R*-factors based on *F*^2^ are statistically about twice as large as those based on *F*, and *R*- factors based on ALL data will be even larger.

### Fractional atomic coordinates and isotropic or equivalent isotropic displacement parameters (Å^2^)

**Table:**

	*x*	*y*	*z*	*U*_iso_*/*U*_eq_	
Co1	0.843849 (18)	0.27327 (4)	0.14261 (12)	0.00806 (7)	
N11	0.77135 (12)	0.0306 (3)	0.11765 (9)	0.0119 (3)	
H11A	0.7676 (16)	−0.041 (4)	0.1502 (12)	0.018*	
H11B	0.7919 (17)	−0.035 (4)	0.0915 (12)	0.018*	
N12	0.72646 (12)	0.4084 (3)	0.13789 (9)	0.0118 (3)	
H12A	0.7131 (16)	0.487 (4)	0.1049 (12)	0.018*	
H12B	0.7233 (17)	0.484 (4)	0.1675 (13)	0.018*	
C11	0.67584 (13)	0.0895 (3)	0.09310 (10)	0.0151 (4)	
H11C	0.6338	−0.0243	0.096	0.018*	
H11D	0.6718	0.1326	0.0471	0.018*	
C12	0.65116 (14)	0.2597 (3)	0.13567 (10)	0.0147 (4)	
H12C	0.5926	0.3207	0.1172	0.018*	
H12D	0.6448	0.2109	0.1801	0.018*	
N13	0.95848 (12)	0.1259 (3)	0.14299 (8)	0.0112 (3)	
H13A	0.9998 (17)	0.194 (4)	0.1646 (12)	0.017*	
H13B	0.9546 (16)	0.009 (4)	0.1610 (12)	0.017*	
N14	0.85766 (12)	0.3359 (3)	0.05090 (8)	0.0112 (3)	
H14A	0.8164 (18)	0.268 (3)	0.0203 (12)	0.017*	
H14B	0.8437 (16)	0.459 (4)	0.0468 (12)	0.017*	
C13	0.98149 (14)	0.1016 (3)	0.07545 (9)	0.0121 (4)	
H13C	0.9484	−0.0139	0.0542	0.015*	
H13D	1.0479	0.079	0.0759	0.015*	
C14	0.95313 (14)	0.2919 (3)	0.03914 (10)	0.0135 (4)	
H14C	0.994	0.4029	0.0552	0.016*	
H14D	0.9565	0.2746	−0.008	0.016*	
N15	0.83969 (11)	0.2115 (2)	0.23255 (8)	0.0114 (3)	
O11	0.79655 (11)	0.3185 (2)	0.26709 (7)	0.0223 (3)	
O12	0.88006 (10)	0.0626 (2)	0.25612 (7)	0.0182 (3)	
N16	0.91005 (11)	0.5117 (2)	0.16892 (8)	0.0121 (3)	
O13	0.88884 (11)	0.6728 (2)	0.14137 (7)	0.0195 (3)	
O14	0.97386 (10)	0.5019 (2)	0.21304 (7)	0.0198 (3)	
Co2	0.300637 (18)	0.21539 (4)	0.116445 (12)	0.00813 (7)	
N21	0.34669 (12)	0.1925 (2)	0.20883 (8)	0.0100 (3)	
H21A	0.3033 (18)	0.207 (4)	0.2293 (12)	0.015*	
H21B	0.3695 (16)	0.076 (4)	0.2176 (11)	0.015*	
N22	0.35638 (12)	0.4797 (2)	0.12156 (8)	0.0112 (3)	
H22A	0.3196 (17)	0.563 (4)	0.1050 (12)	0.017*	
H22B	0.4061 (17)	0.477 (4)	0.1013 (11)	0.017*	
C21	0.41547 (14)	0.3501 (3)	0.22704 (10)	0.0127 (4)	
H21C	0.4755	0.3103	0.2145	0.015*	
H21D	0.4222	0.3735	0.2746	0.015*	
C22	0.38153 (14)	0.5361 (3)	0.19102 (9)	0.0134 (4)	
H22C	0.3277	0.59	0.2094	0.016*	
H22D	0.4299	0.6393	0.1948	0.016*	
N23	0.18840 (11)	0.3367 (2)	0.13875 (8)	0.0116 (3)	
O21	0.14792 (10)	0.4608 (2)	0.10178 (7)	0.0159 (3)	
O22	0.15768 (11)	0.2921 (2)	0.19042 (7)	0.0191 (3)	
N24	0.25641 (12)	0.2582 (2)	0.02614 (8)	0.0117 (3)	
O23	0.18335 (10)	0.1818 (2)	0.00146 (7)	0.0176 (3)	
O24	0.29753 (10)	0.3733 (2)	−0.00703 (7)	0.0167 (3)	
N25	0.41370 (11)	0.0952 (2)	0.09661 (8)	0.0114 (3)	
O25	0.44644 (10)	−0.0461 (2)	0.13136 (7)	0.0180 (3)	
O26	0.45432 (10)	0.1533 (2)	0.05169 (7)	0.0186 (3)	
N26	0.25080 (11)	−0.0494 (2)	0.11586 (8)	0.0130 (3)	
O27	0.25612 (10)	−0.1596 (2)	0.06758 (7)	0.0172 (3)	
O28	0.22021 (11)	−0.1164 (2)	0.16421 (7)	0.0201 (3)	
O1	0.52233 (12)	0.6237 (2)	0.06827 (8)	0.0216 (3)	
H1A	0.5386 (19)	0.649 (4)	0.0315 (14)	0.032*	
H1B	0.511 (2)	0.731 (4)	0.0847 (15)	0.032*	

### Atomic displacement parameters (Å^2^)

**Table:**

	*U*^11^	*U*^22^	*U*^33^	*U*^12^	*U*^13^	*U*^23^
Co1	0.00877 (13)	0.00827 (12)	0.00724 (13)	0.00044 (9)	0.00131 (9)	−0.00040 (9)
N11	0.0140 (9)	0.0097 (8)	0.0128 (8)	−0.0006 (6)	0.0045 (7)	−0.0011 (6)
N12	0.0121 (8)	0.0131 (8)	0.0104 (8)	0.0025 (6)	0.0013 (6)	−0.0011 (6)
C11	0.0100 (9)	0.0163 (10)	0.0190 (10)	−0.0008 (7)	0.0008 (8)	−0.0046 (8)
C12	0.0114 (10)	0.0156 (10)	0.0177 (10)	−0.0004 (7)	0.0042 (8)	−0.0025 (7)
N13	0.0100 (8)	0.0123 (8)	0.0108 (8)	0.0016 (6)	−0.0007 (6)	−0.0006 (6)
N14	0.0121 (8)	0.0118 (8)	0.0099 (8)	0.0007 (6)	0.0023 (6)	0.0013 (6)
C13	0.0107 (9)	0.0137 (9)	0.0122 (9)	−0.0001 (7)	0.0026 (7)	−0.0021 (7)
C14	0.0140 (10)	0.0143 (9)	0.0130 (9)	−0.0004 (7)	0.0045 (8)	0.0000 (7)
N15	0.0098 (8)	0.0138 (8)	0.0106 (8)	−0.0001 (6)	0.0015 (6)	−0.0001 (6)
O11	0.0270 (9)	0.0296 (9)	0.0117 (7)	0.0126 (7)	0.0076 (6)	−0.0005 (6)
O12	0.0220 (8)	0.0173 (7)	0.0152 (7)	0.0047 (6)	0.0021 (6)	0.0068 (6)
N16	0.0129 (8)	0.0124 (8)	0.0111 (8)	−0.0001 (6)	0.0018 (6)	−0.0023 (6)
O13	0.0266 (9)	0.0096 (7)	0.0215 (8)	0.0006 (6)	0.0003 (6)	0.0032 (6)
O14	0.0183 (8)	0.0192 (8)	0.0195 (8)	−0.0024 (6)	−0.0076 (6)	−0.0027 (6)
Co2	0.00897 (13)	0.00782 (12)	0.00761 (13)	−0.00003 (9)	0.00110 (9)	0.00019 (9)
N21	0.0111 (8)	0.0100 (8)	0.0091 (8)	0.0008 (6)	0.0016 (6)	0.0010 (6)
N22	0.0118 (8)	0.0093 (8)	0.0128 (8)	0.0016 (6)	0.0025 (7)	0.0010 (6)
C21	0.0128 (10)	0.0122 (9)	0.0123 (9)	−0.0020 (7)	−0.0013 (7)	0.0000 (7)
C22	0.0166 (10)	0.0117 (9)	0.0118 (9)	−0.0011 (7)	0.0010 (8)	−0.0034 (7)
N23	0.0089 (8)	0.0116 (8)	0.0141 (8)	−0.0014 (6)	0.0012 (6)	−0.0014 (6)
O21	0.0160 (7)	0.0135 (7)	0.0179 (7)	0.0043 (6)	0.0001 (6)	0.0022 (6)
O22	0.0174 (8)	0.0271 (8)	0.0143 (7)	0.0045 (6)	0.0077 (6)	0.0043 (6)
N24	0.0137 (8)	0.0123 (8)	0.0094 (8)	0.0022 (6)	0.0020 (6)	−0.0006 (6)
O23	0.0141 (7)	0.0230 (8)	0.0146 (7)	−0.0013 (6)	−0.0019 (6)	−0.0029 (6)
O24	0.0182 (8)	0.0199 (7)	0.0124 (7)	0.0016 (6)	0.0038 (6)	0.0058 (6)
N25	0.0127 (8)	0.0097 (7)	0.0115 (8)	−0.0014 (6)	0.0003 (6)	−0.0019 (6)
O25	0.0209 (8)	0.0145 (7)	0.0192 (8)	0.0079 (6)	0.0045 (6)	0.0053 (6)
O26	0.0168 (8)	0.0231 (8)	0.0172 (8)	0.0036 (6)	0.0076 (6)	0.0047 (6)
N26	0.0109 (8)	0.0123 (8)	0.0152 (8)	−0.0003 (6)	−0.0014 (6)	−0.0004 (6)
O27	0.0211 (8)	0.0130 (7)	0.0168 (7)	−0.0019 (6)	−0.0007 (6)	−0.0057 (6)
O28	0.0257 (8)	0.0172 (8)	0.0185 (8)	−0.0058 (6)	0.0066 (6)	0.0031 (6)
O1	0.0287 (9)	0.0147 (8)	0.0240 (9)	0.0005 (7)	0.0139 (7)	0.0016 (6)

### Geometric parameters (Å, º)

**Table:**

Co1—N15	1.9141 (17)	N16—O14	1.231 (2)
Co1—N16	1.9177 (16)	N16—O13	1.244 (2)
Co1—N12	1.9471 (17)	Co2—N26	1.9215 (17)
Co1—N13	1.9586 (17)	Co2—N24	1.9240 (17)
Co1—N14	1.9775 (17)	Co2—N25	1.9413 (17)
Co1—N11	1.9811 (17)	Co2—N23	1.9502 (16)
N11—C11	1.492 (3)	Co2—N22	1.9512 (17)
N11—H11A	0.83 (3)	Co2—N21	1.9551 (17)
N11—H11B	0.79 (3)	N21—C21	1.482 (3)
N12—C12	1.490 (3)	N21—H21A	0.82 (3)
N12—H12A	0.86 (3)	N21—H21B	0.86 (2)
N12—H12B	0.80 (3)	N22—C22	1.489 (2)
C11—C12	1.513 (3)	N22—H22A	0.83 (2)
C11—H11C	0.99	N22—H22B	0.89 (2)
C11—H11D	0.99	C21—C22	1.507 (3)
C12—H12C	0.99	C21—H21C	0.99
C12—H12D	0.99	C21—H21D	0.99
N13—C13	1.486 (2)	C22—H22C	0.99
N13—H13A	0.84 (3)	C22—H22D	0.99
N13—H13B	0.87 (3)	N23—O21	1.234 (2)
N14—C14	1.488 (3)	N23—O22	1.247 (2)
N14—H14A	0.94 (3)	N24—O24	1.239 (2)
N14—H14B	0.85 (2)	N24—O23	1.246 (2)
C13—C14	1.514 (3)	N25—O26	1.229 (2)
C13—H13C	0.99	N25—O25	1.250 (2)
C13—H13D	0.99	N26—O28	1.231 (2)
C14—H14C	0.99	N26—O27	1.252 (2)
C14—H14D	0.99	O1—H1A	0.84 (3)
N15—O12	1.233 (2)	O1—H1B	0.82 (3)
N15—O11	1.241 (2)		
			
N15—Co1—N16	88.84 (7)	C13—C14—H14D	110.3
N15—Co1—N12	90.93 (7)	H14C—C14—H14D	108.5
N16—Co1—N12	92.62 (7)	O12—N15—O11	119.85 (17)
N15—Co1—N13	90.95 (7)	O12—N15—Co1	119.25 (13)
N16—Co1—N13	90.55 (7)	O11—N15—Co1	120.90 (13)
N12—Co1—N13	176.35 (7)	O14—N16—O13	120.87 (17)
N15—Co1—N14	175.95 (7)	O14—N16—Co1	118.93 (13)
N16—Co1—N14	89.24 (7)	O13—N16—Co1	120.20 (13)
N12—Co1—N14	92.73 (7)	N26—Co2—N24	92.70 (7)
N13—Co1—N14	85.50 (7)	N26—Co2—N25	87.31 (7)
N15—Co1—N11	89.76 (7)	N24—Co2—N25	93.20 (7)
N16—Co1—N11	177.57 (7)	N26—Co2—N23	92.95 (7)
N12—Co1—N11	85.42 (7)	N24—Co2—N23	88.26 (7)
N13—Co1—N11	91.46 (7)	N25—Co2—N23	178.50 (7)
N14—Co1—N11	92.28 (7)	N26—Co2—N22	176.56 (7)
C11—N11—Co1	109.24 (12)	N24—Co2—N22	90.48 (7)
C11—N11—H11A	105.9 (17)	N25—Co2—N22	91.21 (7)
Co1—N11—H11A	110.3 (17)	N23—Co2—N22	88.45 (7)
C11—N11—H11B	109.9 (18)	N26—Co2—N21	91.20 (7)
Co1—N11—H11B	113.5 (18)	N24—Co2—N21	175.92 (7)
H11A—N11—H11B	108 (2)	N25—Co2—N21	88.17 (7)
C12—N12—Co1	110.22 (13)	N23—Co2—N21	90.34 (7)
C12—N12—H12A	106.5 (16)	N22—Co2—N21	85.65 (7)
Co1—N12—H12A	116.0 (17)	C21—N21—Co2	109.77 (12)
C12—N12—H12B	109.5 (18)	C21—N21—H21A	109.7 (17)
Co1—N12—H12B	113.0 (18)	Co2—N21—H21A	107.2 (18)
H12A—N12—H12B	101 (2)	C21—N21—H21B	110.9 (15)
N11—C11—C12	106.59 (16)	Co2—N21—H21B	110.9 (15)
N11—C11—H11C	110.4	H21A—N21—H21B	108 (2)
C12—C11—H11C	110.4	C22—N22—Co2	109.71 (12)
N11—C11—H11D	110.4	C22—N22—H22A	107.6 (17)
C12—C11—H11D	110.4	Co2—N22—H22A	110.1 (17)
H11C—C11—H11D	108.6	C22—N22—H22B	109.7 (15)
N12—C12—C11	106.97 (17)	Co2—N22—H22B	108.8 (16)
N12—C12—H12C	110.3	H22A—N22—H22B	111 (2)
C11—C12—H12C	110.3	N21—C21—C22	106.76 (15)
N12—C12—H12D	110.3	N21—C21—H21C	110.4
C11—C12—H12D	110.3	C22—C21—H21C	110.4
H12C—C12—H12D	108.6	N21—C21—H21D	110.4
C13—N13—Co1	110.33 (12)	C22—C21—H21D	110.4
C13—N13—H13A	108.9 (17)	H21C—C21—H21D	108.6
Co1—N13—H13A	107.4 (17)	N22—C22—C21	107.21 (15)
C13—N13—H13B	109.6 (16)	N22—C22—H22C	110.3
Co1—N13—H13B	110.8 (16)	C21—C22—H22C	110.3
H13A—N13—H13B	110 (2)	N22—C22—H22D	110.3
C14—N14—Co1	108.93 (12)	C21—C22—H22D	110.3
C14—N14—H14A	110.0 (16)	H22C—C22—H22D	108.5
Co1—N14—H14A	114.1 (15)	O21—N23—O22	119.58 (16)
C14—N14—H14B	113.5 (17)	O21—N23—Co2	119.71 (13)
Co1—N14—H14B	104.5 (16)	O22—N23—Co2	120.69 (13)
H14A—N14—H14B	106 (2)	O24—N24—O23	119.14 (16)
N13—C13—C14	107.04 (15)	O24—N24—Co2	119.94 (13)
N13—C13—H13C	110.3	O23—N24—Co2	120.75 (13)
C14—C13—H13C	110.3	O26—N25—O25	119.03 (16)
N13—C13—H13D	110.3	O26—N25—Co2	122.59 (13)
C14—C13—H13D	110.3	O25—N25—Co2	118.38 (13)
H13C—C13—H13D	108.6	O28—N26—O27	119.82 (17)
N14—C14—C13	107.25 (16)	O28—N26—Co2	120.66 (13)
N14—C14—H14C	110.3	O27—N26—Co2	119.29 (14)
C13—C14—H14C	110.3	H1A—O1—H1B	107 (3)
N14—C14—H14D	110.3		
			
N15—Co1—N11—C11	105.28 (14)	N22—Co2—N21—C21	−14.82 (13)
N12—Co1—N11—C11	14.33 (14)	N24—Co2—N22—C22	165.62 (13)
N13—Co1—N11—C11	−163.77 (14)	N25—Co2—N22—C22	−101.17 (13)
N14—Co1—N11—C11	−78.22 (14)	N23—Co2—N22—C22	77.37 (13)
N15—Co1—N12—C12	−75.67 (14)	N21—Co2—N22—C22	−13.09 (13)
N16—Co1—N12—C12	−164.55 (14)	Co2—N21—C21—C22	38.90 (18)
N14—Co1—N12—C12	106.08 (14)	Co2—N22—C22—C21	37.65 (18)
N11—Co1—N12—C12	14.01 (14)	N26—Co2—N23—O21	−120.29 (15)
Co1—N11—C11—C12	−38.82 (19)	N24—Co2—N23—O21	−27.67 (15)
Co1—N12—C12—C11	−38.91 (19)	N22—Co2—N23—O21	62.86 (15)
N15—Co1—N13—C13	169.58 (13)	N21—Co2—N23—O21	148.49 (15)
N16—Co1—N13—C13	−101.57 (13)	N26—Co2—N23—O22	60.64 (16)
N14—Co1—N13—C13	−12.38 (13)	N24—Co2—N23—O22	153.25 (16)
N11—Co1—N13—C13	79.80 (13)	N22—Co2—N23—O22	−116.22 (16)
N16—Co1—N14—C14	75.08 (13)	N21—Co2—N23—O22	−30.58 (16)
N12—Co1—N14—C14	167.66 (13)	N26—Co2—N24—O24	−151.60 (15)
N13—Co1—N14—C14	−15.54 (13)	N25—Co2—N24—O24	−64.15 (15)
N11—Co1—N14—C14	−106.82 (13)	N23—Co2—N24—O24	115.53 (15)
Co1—N13—C13—C14	37.01 (18)	N22—Co2—N24—O24	27.10 (15)
Co1—N14—C14—C13	39.52 (18)	N26—Co2—N24—O23	33.20 (15)
N16—Co1—N15—O12	−114.95 (15)	N25—Co2—N24—O23	120.65 (15)
N12—Co1—N15—O12	152.45 (15)	N23—Co2—N24—O23	−59.66 (15)
N13—Co1—N15—O12	−24.42 (15)	N22—Co2—N24—O23	−148.10 (15)
N11—Co1—N15—O12	67.03 (15)	N26—Co2—N25—O26	129.51 (16)
N16—Co1—N15—O11	65.51 (16)	N24—Co2—N25—O26	36.95 (16)
N12—Co1—N15—O11	−27.09 (16)	N22—Co2—N25—O26	−53.60 (16)
N13—Co1—N15—O11	156.04 (16)	N21—Co2—N25—O26	−139.21 (16)
N11—Co1—N15—O11	−112.51 (16)	N26—Co2—N25—O25	−50.98 (14)
N15—Co1—N16—O14	45.77 (15)	N24—Co2—N25—O25	−143.54 (14)
N12—Co1—N16—O14	136.64 (15)	N22—Co2—N25—O25	125.91 (14)
N13—Co1—N16—O14	−45.17 (15)	N21—Co2—N25—O25	40.30 (14)
N14—Co1—N16—O14	−130.66 (15)	N24—Co2—N26—O28	−145.07 (15)
N15—Co1—N16—O13	−134.66 (15)	N25—Co2—N26—O28	121.85 (15)
N12—Co1—N16—O13	−43.78 (16)	N23—Co2—N26—O28	−56.67 (15)
N13—Co1—N16—O13	134.40 (15)	N21—Co2—N26—O28	33.73 (16)
N14—Co1—N16—O13	48.91 (15)	N24—Co2—N26—O27	40.49 (15)
N26—Co2—N21—C21	163.81 (13)	N25—Co2—N26—O27	−52.59 (15)
N25—Co2—N21—C21	76.54 (13)	N23—Co2—N26—O27	128.89 (15)
N23—Co2—N21—C21	−103.24 (13)	N21—Co2—N26—O27	−140.70 (15)

### Hydrogen-bond geometry (Å, º)

**Table:**

*D*—H···*A*	*D*—H	H···*A*	*D*···*A*	*D*—H···*A*
N11—H11*A*···O11^i^	0.83 (3)	2.26 (3)	3.048 (2)	158 (2)
N11—H11*B*···O23^ii^	0.79 (3)	2.23 (3)	2.991 (2)	163 (2)
N11—H11*B*···O13^iii^	0.79 (3)	2.57 (2)	2.966 (2)	113 (2)
N12—H12*A*···O24^iv^	0.86 (3)	2.22 (3)	3.060 (2)	164 (2)
N12—H12*B*···O12^v^	0.80 (3)	2.38 (3)	3.030 (2)	139 (2)
N13—H13*A*···O22^vi^	0.84 (3)	2.42 (3)	3.186 (2)	152 (2)
N13—H13*B*···O13^iii^	0.87 (3)	2.47 (3)	3.206 (2)	143 (2)
N14—H14*A*···O27^ii^	0.94 (3)	2.12 (3)	3.038 (2)	164 (2)
N14—H14*B*···O24^iv^	0.85 (2)	2.42 (2)	3.060 (2)	132 (2)
N21—H21*A*···O28^vii^	0.82 (3)	2.56 (3)	3.185 (2)	134 (2)
N22—H22*A*···O27^viii^	0.83 (2)	2.18 (3)	2.981 (2)	164 (2)
N22—H22*B*···O1	0.89 (2)	2.16 (3)	2.967 (2)	151 (2)
O1—H1*A*···O26^iv^	0.84 (3)	2.19 (3)	2.953 (2)	152 (3)
O1—H1*A*···O24^iv^	0.84 (3)	2.53 (3)	3.081 (2)	124 (2)
O1—H1*B*···O25^viii^	0.82 (3)	2.07 (3)	2.866 (2)	162 (3)

Symmetry codes: (i) −*x*+3/2, *y*−1/2, −*z*+1/2; (ii) −*x*+1, −*y*, −*z*; (iii) *x*, *y*−1, *z*; (iv) −*x*+1, −*y*+1, −*z*; (v) −*x*+3/2, *y*+1/2, −*z*+1/2; (vi) *x*+1, *y*, *z*; (vii) −*x*+1/2, *y*+1/2, −*z*+1/2; (viii) *x*, *y*+1, *z*.
